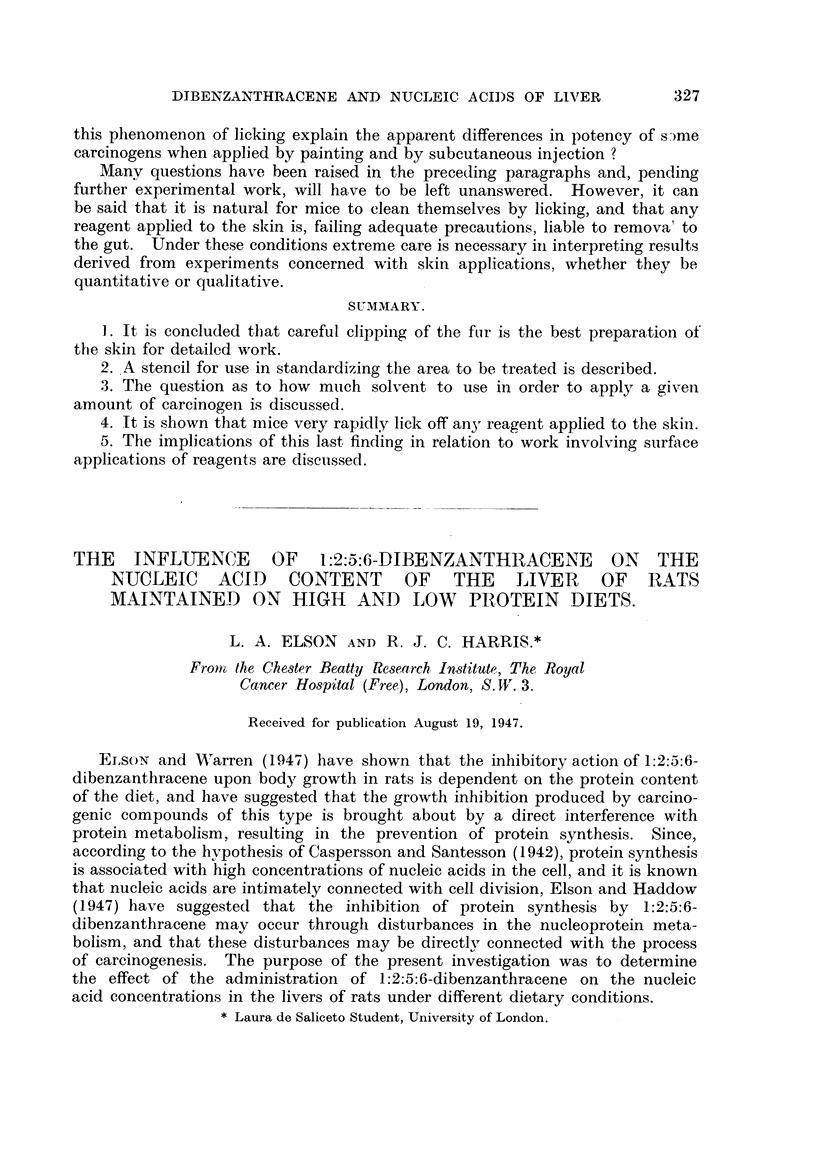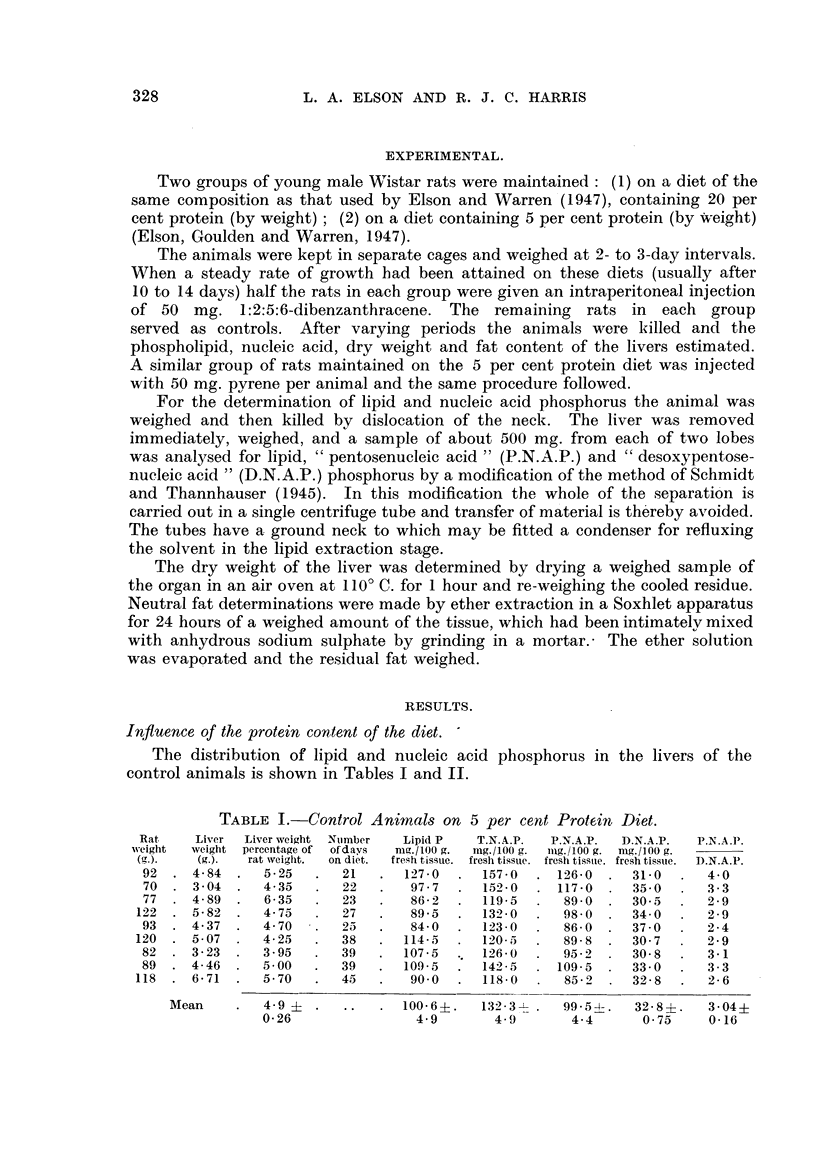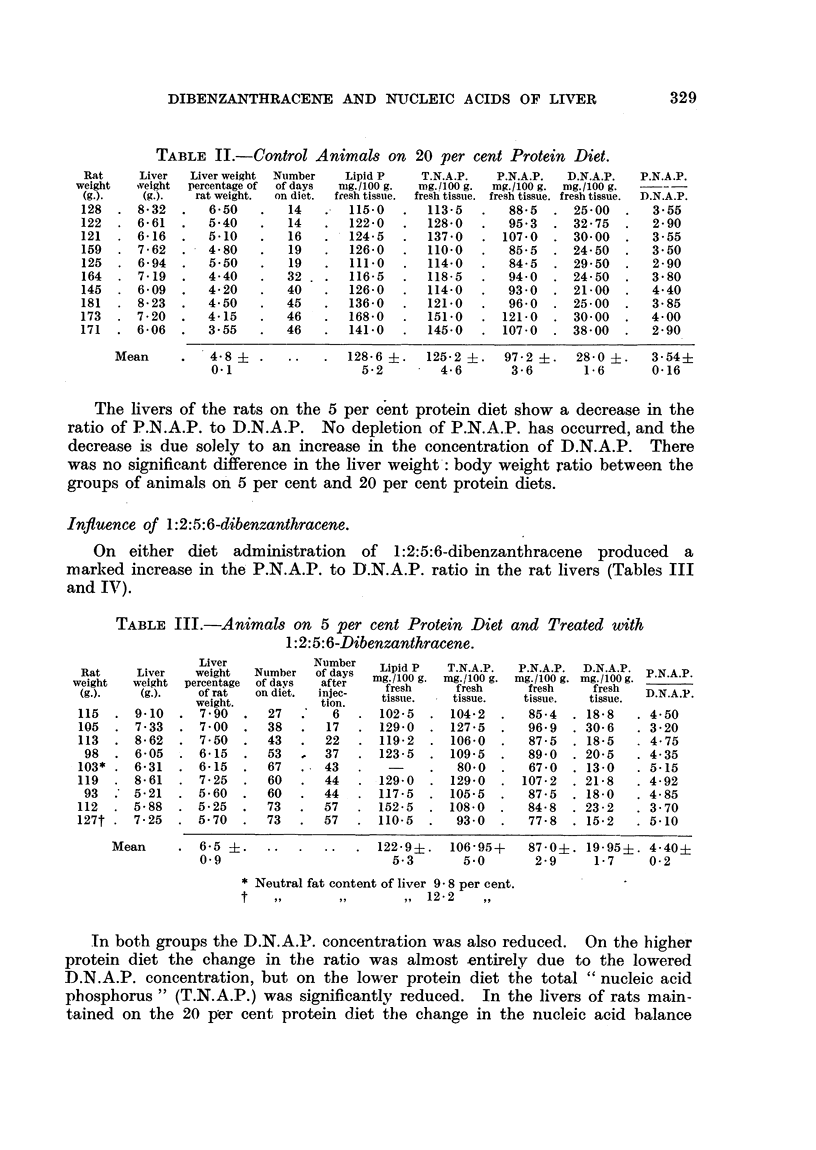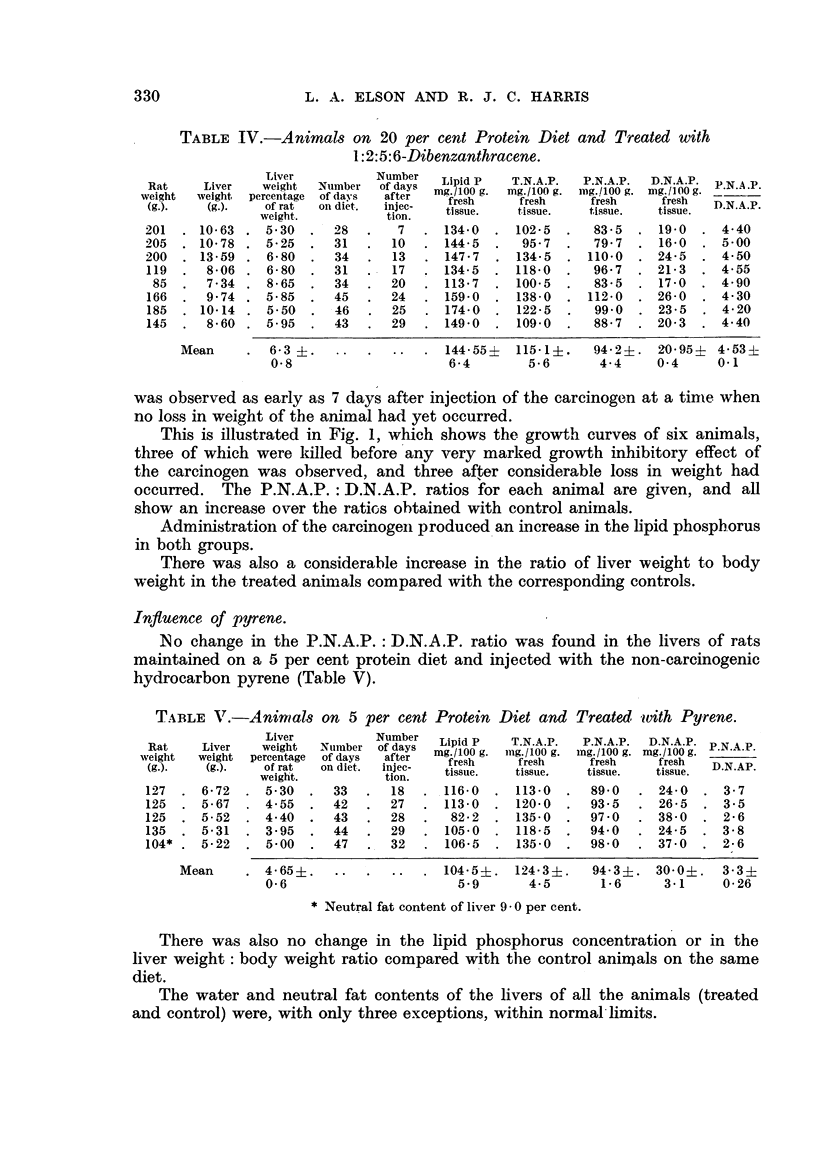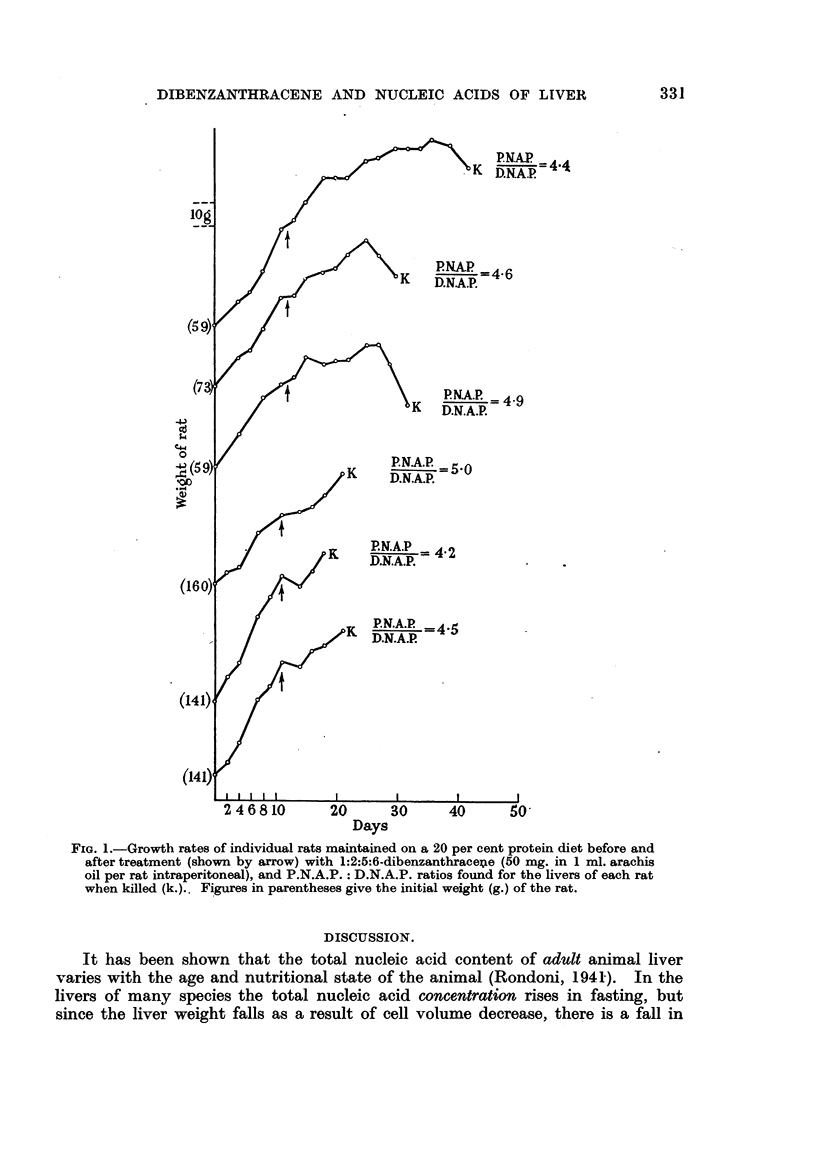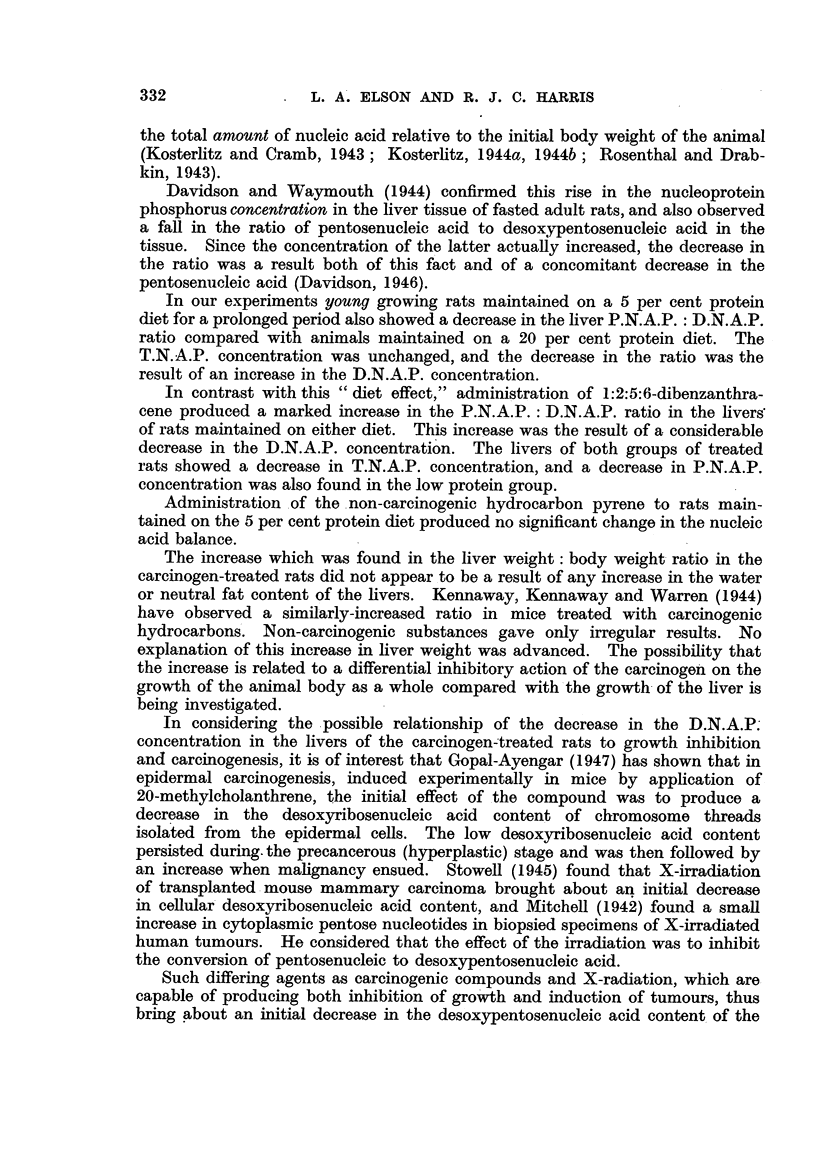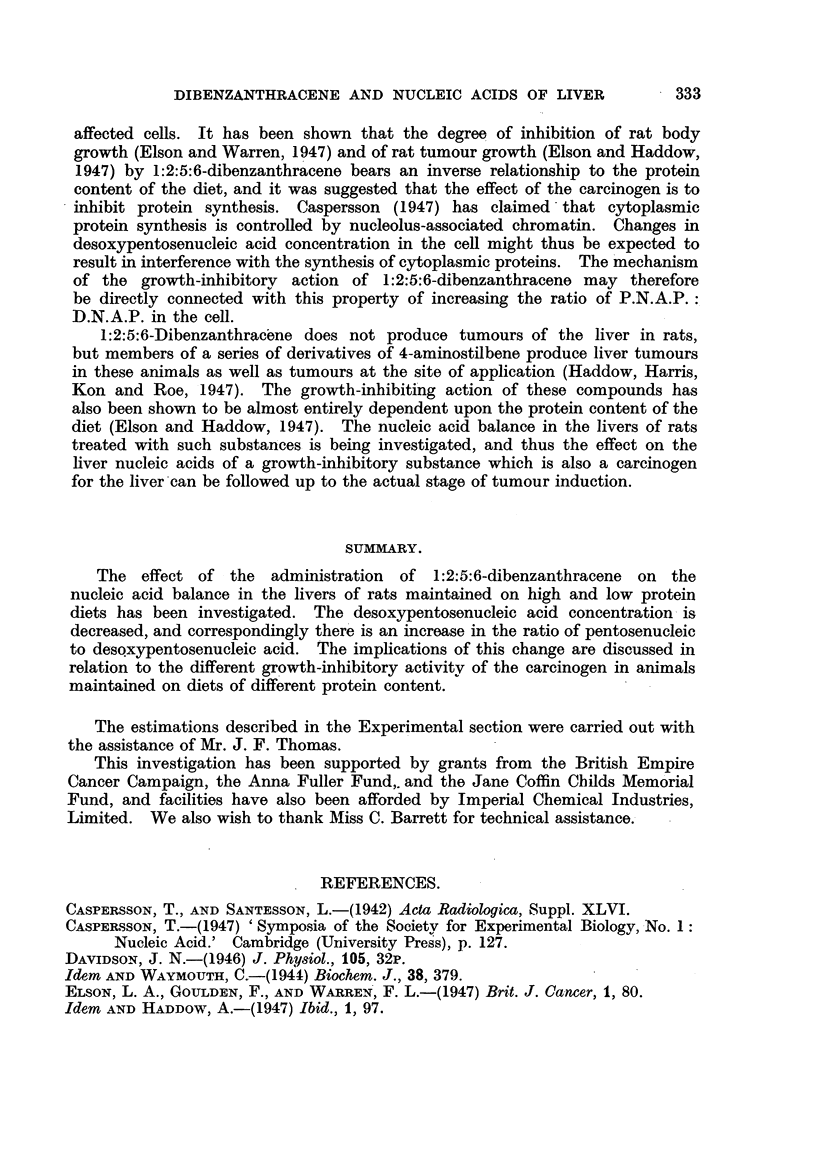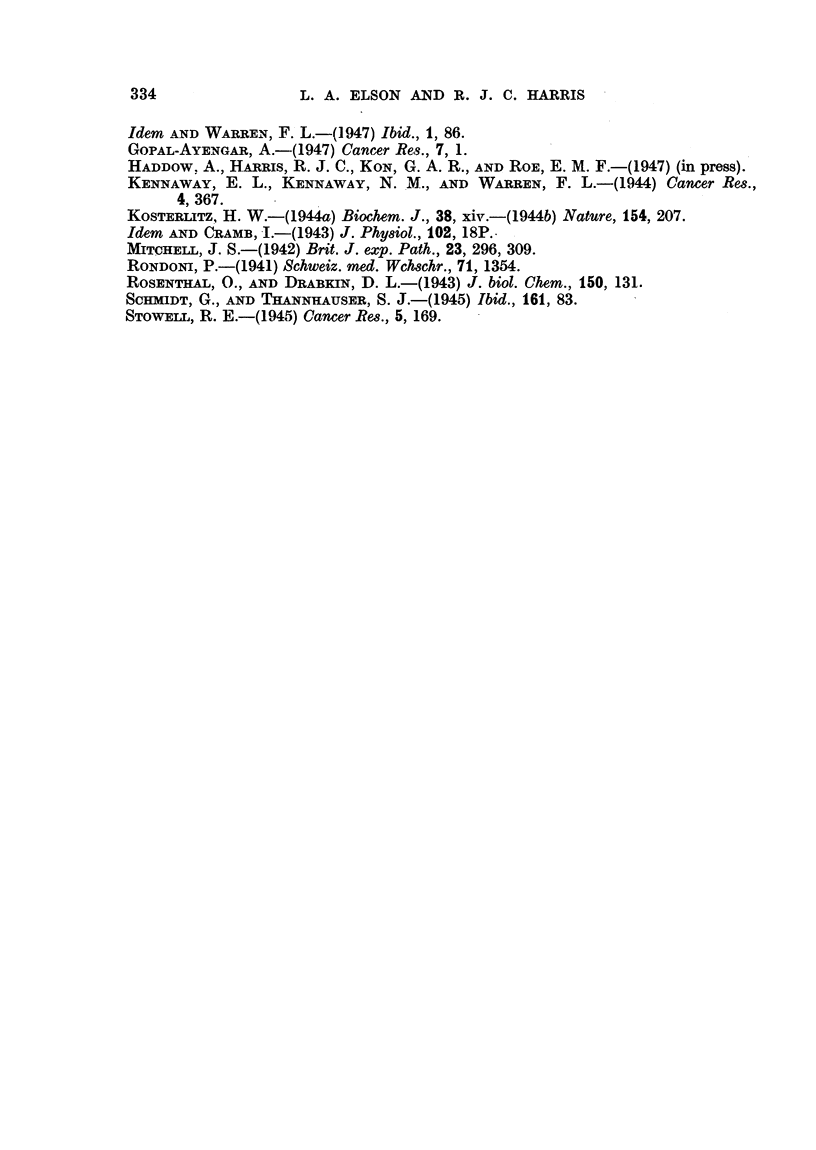# The Influence of 1:2:5:6-Dibenzanthracene on the Nucleic Acid Content of the Liver of Rats Maintained on High and Low Protein Diets

**DOI:** 10.1038/bjc.1947.29

**Published:** 1947-09

**Authors:** L. A. Elson, R. J. C. Harris


### THE INFLUENCE OF 1:2:5:6-DIBENZANTHRACENE ON THE NUCLEIC ACID CONTENT OF THE LIVER OF RATS MAINTAINED ON HIGH AND LOW PROTEIN DIETS.

L. A. ELSON AND R. J. C. HARRIS.*

*From the Chester Beatty Research Institute, The Royal Cancer Hospital (Free), London, S.W. 3.*

Received for publication August 19, 1947.

Elson and Warren (1947) have shown that the inhibitory action of 1:2:5:6-dibenzanthracene upon body growth in rats is dependent on the protein content of the diet, and have suggested that the growth inhibition produced by carcinogenic compounds of this type is brought about by a direct interference with protein metabolism, resulting in the prevention of protein synthesis. Since, according to the hypothesis of Caspersson and Santesson (1942), protein synthesis is associated with high concentrations of nucleic acids in the cell, and it is known that nucleic acids are intimately connected with cell division, Elson and Haddow (1947) have suggested that the inhibition of protein synthesis by 1:2:5:6-dibenzanthracene may occur through disturbances in the nucleoprotein metabolism, and that these disturbances may be directly connected with the process of carcinogenesis. The purpose of the present investigation was to determine the effect of the administration of 1:2:5:6-dibenzanthracene on the nucleic acid concentrations in the livers of rats under different dietary conditions.

* Laura de Saliceto Student, University of London.

### EXPERIMENTAL.

Two groups of young male Wistar rats were maintained : (1) on a diet of the same composition as that used by Elson and Warren (1947), containing 20 per cent protein (by weight) ; (2) on a diet containing 5 per cent protein (by weight) (Elson, Goulden and Warren, 1947).

The animals were kept in separate cages and weighed at 2- to 3-day intervals. When a steady rate of growth had been attained on these diets (usually after 10 to 14 days) half the rats in each group were given an intraperitoneal injection of 50 mg. 1:2:5:6-dibenzanthracene. The remaining rats in each group served as controls. After varying periods the animals were killed and the phospholipid, nucleic acid, dry weight and fat content of the livers estimated. A similar group of rats maintained on the 5 per cent protein diet was injected with 50 mg. pyrene per animal and the same procedure followed.

For the determination of lipid and nucleic acid phosphorus the animal was weighed and then killed by dislocation of the neck. The liver was removed immediately, weighed, and a sample of about 500 mg. from each of two lobes was analysed for lipid, " pentosenucleic acid " (P.N.A.P.) and " desoxypentose-nucleic acid " (D.N.A.P.) phosphorus by a modification of the method of Schmidt and Thannhauser (1945). In this modification the whole of the separation is carried out in a single centrifuge tube and transfer of material is thereby avoided. The tubes have a ground neck to which may be fitted a condenser for refluxing the solvent in the lipid extraction stage.

The dry weight of the liver was determined by drying a weighed sample of the organ in an air oven at 110° C. for 1 hour and re-weighing the cooled residue. Neutral fat determinations were made by ether extraction in a Soxhlet apparatus for 24 hours of a weighed amount of the tissue, which had been intimately mixed with anhydrous sodium sulphate by grinding in a mortar. The ether solution was evaporated and the residual fat weighed.

### RESULTS.

*Influence of the protein content of the diet.*

The distribution of lipid and nucleic acid phosphorus in the livers of the control animals is shown in Tables I and II.

TABLE I.—*Control Animals on 5 per cent Protein Diet.*

| Rat weight (g.). | Liver weight (g.). | Liver weight percentage of rat weight. | Number of days on diet. | Lipid P mg./100 g. fresh tissue. | T.N.A.P. mg./100 g. fresh tissue. | P.N.A.P. mg./100 g. fresh tissue. | D.N.A.P. mg./100 g. fresh tissue. | P.N.A.P. / D.N.A.P. |
|---|---|---|---|---|---|---|---|---|
| 92 | 4·84 | 5·25 | 21 | 127·0 | 157·0 | 126·0 | 31·0 | 4·0 |
| 70 | 3·04 | 4·35 | 22 | 97·7 | 152·0 | 117·0 | 35·0 | 3·3 |
| 77 | 4·89 | 6·35 | 23 | 86·2 | 119·5 | 89·0 | 30·5 | 2·9 |
| 122 | 5·82 | 4·75 | 27 | 89·5 | 132·0 | 98·0 | 34·0 | 2·9 |
| 93 | 4·37 | 4·70 | 25 | 84·0 | 123·0 | 86·0 | 37·0 | 2·4 |
| 120 | 5·07 | 4·25 | 38 | 114·5 | 120·5 | 89·8 | 30·7 | 2·9 |
| 82 | 3·23 | 3·95 | 39 | 107·5 | 126·0 | 95·2 | 30·8 | 3·1 |
| 89 | 4·46 | 5·00 | 39 | 109·5 | 142·5 | 109·5 | 33·0 | 3·3 |
| 118 | 6·71 | 5·70 | 45 | 90·0 | 118·0 | 85·2 | 32·8 | 2·6 |
| Mean | | 4·9 ± 0·26 | .. | 100·6 ± 4·9 | 132·3 ± 4·9 | 99·5 ± 4·4 | 32·8 ± 0·75 | 3·04 ± 0·16 |

TABLE II.—*Control Animals on 20 per cent Protein Diet.*

| Rat weight (g.). | Liver weight (g.). | Liver weight percentage of rat weight. | Number of days on diet. | Lipid P mg./100 g. fresh tissue. | T.N.A.P. mg./100 g. fresh tissue. | P.N.A.P. mg./100 g. fresh tissue. | D.N.A.P. mg./100 g. fresh tissue. | P.N.A.P. / D.N.A.P. |
|---|---|---|---|---|---|---|---|---|
| 128 | 8·32 | 6·50 | 14 | 115·0 | 113·5 | 88·5 | 25·00 | 3·55 |
| 122 | 6·61 | 5·40 | 14 | 122·0 | 128·0 | 95·3 | 32·75 | 2·90 |
| 121 | 6·16 | 5·10 | 16 | 124·5 | 137·0 | 107·0 | 30·00 | 3·55 |
| 159 | 7·62 | 4·80 | 19 | 126·0 | 110·0 | 85·5 | 24·50 | 3·50 |
| 125 | 6·94 | 5·50 | 19 | 111·0 | 114·0 | 84·5 | 29·50 | 2·90 |
| 164 | 7·19 | 4·40 | 32 | 116·5 | 118·5 | 94·0 | 24·50 | 3·80 |
| 145 | 6·09 | 4·20 | 40 | 126·0 | 114·0 | 93·0 | 21·00 | 4·40 |
| 181 | 8·23 | 4·50 | 45 | 136·0 | 121·0 | 96·0 | 25·00 | 3·85 |
| 173 | 7·20 | 4·15 | 46 | 168·0 | 151·0 | 121·0 | 30·00 | 4·00 |
| 171 | 6·06 | 3·55 | 46 | 141·0 | 145·0 | 107·0 | 38·00 | 2·90 |
| Mean | | 4·8 ± 0·1 | .. | 128·6 ± 5·2 | 125·2 ± 4·6 | 97·2 ± 3·6 | 28·0 ± 1·6 | 3·54 ± 0·16 |

The livers of the rats on the 5 per cent protein diet show a decrease in the ratio of P.N.A.P. to D.N.A.P. No depletion of P.N.A.P. has occurred, and the decrease is due solely to an increase in the concentration of D.N.A.P. There was no significant difference in the liver weight : body weight ratio between the groups of animals on 5 per cent and 20 per cent protein diets.

*Influence of* 1:2:5:6-*dibenzanthracene.*

On either diet administration of 1:2:5:6-dibenzanthracene produced a marked increase in the P.N.A.P. to D.N.A.P. ratio in the rat livers (Tables III and IV).

TABLE III.—*Animals on 5 per cent Protein Diet and Treated with 1:2:5:6-Dibenzanthracene.*

| Rat weight (g.). | Liver weight (g.). | Liver weight percentage of rat weight. | Number of days on diet. | Number of days after injection. | Lipid P mg./100 g. fresh tissue. | T.N.A.P. mg./100 g. fresh tissue. | P.N.A.P. mg./100 g. fresh tissue. | D.N.A.P. mg./100 g. fresh tissue. | P.N.A.P. / D.N.A.P. |
|---|---|---|---|---|---|---|---|---|---|
| 115 | 9·10 | 7·90 | 27 | 6 | 102·5 | 104·2 | 85·4 | 18·8 | 4·50 |
| 105 | 7·33 | 7·00 | 38 | 17 | 129·0 | 127·5 | 96·9 | 30·6 | 3·20 |
| 113 | 8·62 | 7·50 | 43 | 22 | 119·2 | 106·0 | 87·5 | 18·5 | 4·75 |
| 98 | 6·05 | 6·15 | 53 | 37 | 123·5 | 109·5 | 89·0 | 20·5 | 4·35 |
| 103* | 6·31 | 6·15 | 67 | 43 | — | 80·0 | 67·0 | 13·0 | 5·15 |
| 119 | 8·61 | 7·25 | 60 | 44 | 129·0 | 129·0 | 107·2 | 21·8 | 4·92 |
| 93 | 5·21 | 5·60 | 60 | 44 | 117·5 | 105·5 | 87·5 | 18·0 | 4·85 |
| 112 | 5·88 | 5·25 | 73 | 57 | 152·5 | 108·0 | 84·8 | 23·2 | 3·70 |
| 127† | 7·25 | 5·70 | 73 | 57 | 110·5 | 93·0 | 77·8 | 15·2 | 5·10 |
| Mean | | 6·5 ± 0·9 | .. | .. | 122·9 ± 5·3 | 106·95 + 5·0 | 87·0 ± 2·9 | 19·95 ± 1·7 | 4·40 ± 0·2 |

* Neutral fat content of liver 9·8 per cent.
† ,, ,, ,, 12·2 ,,

In both groups the D.N.A.P. concentration was also reduced. On the higher protein diet the change in the ratio was almost entirely due to the lowered D.N.A.P. concentration, but on the lower protein diet the total " nucleic acid phosphorus " (T.N.A.P.) was significantly reduced. In the livers of rats maintained on the 20 per cent protein diet the change in the nucleic acid balance

TABLE IV.—*Animals on 20 per cent Protein Diet and Treated with 1:2:5:6-Dibenzanthracene.*

| Rat weight (g.). | Liver weight (g.). | Liver weight percentage of rat weight. | Number of days on diet. | Number of days after injection. | Lipid P mg./100 g. fresh tissue. | T.N.A.P. mg./100 g. fresh tissue. | P.N.A.P. mg./100 g. fresh tissue. | D.N.A.P. mg./100 g. fresh tissue. | P.N.A.P. / D.N.A.P. |
|---|---|---|---|---|---|---|---|---|---|
| 201 | 10·63 | 5·30 | 28 | 7 | 134·0 | 102·5 | 83·5 | 19·0 | 4·40 |
| 205 | 10·78 | 5·25 | 31 | 10 | 144·5 | 95·7 | 79·7 | 16·0 | 5·00 |
| 200 | 13·59 | 6·80 | 34 | 13 | 147·7 | 134·5 | 110·0 | 24·5 | 4·50 |
| 119 | 8·06 | 6·80 | 31 | 17 | 134·5 | 118·0 | 96·7 | 21·3 | 4·55 |
| 85 | 7·34 | 8·65 | 34 | 20 | 113·7 | 100·5 | 83·5 | 17·0 | 4·90 |
| 166 | 9·74 | 5·85 | 45 | 24 | 159·0 | 138·0 | 112·0 | 26·0 | 4·30 |
| 185 | 10·14 | 5·50 | 46 | 25 | 174·0 | 122·5 | 99·0 | 23·5 | 4·20 |
| 145 | 8·60 | 5·95 | 43 | 29 | 149·0 | 109·0 | 88·7 | 20·3 | 4·40 |
| Mean | | 6·3 ± 0·8 | .. | .. | 144·55 ± 6·4 | 115·1 ± 5·6 | 94·2 ± 4·4 | 20·95 ± 0·4 | 4·53 ± 0·1 |

was observed as early as 7 days after injection of the carcinogen at a time when no loss in weight of the animal had yet occurred.

This is illustrated in Fig. 1, which shows the growth curves of six animals, three of which were killed before any very marked growth inhibitory effect of the carcinogen was observed, and three after considerable loss in weight had occurred. The P.N.A.P. : D.N.A.P. ratios for each animal are given, and all show an increase over the ratios obtained with control animals.

Administration of the carcinogen produced an increase in the lipid phosphorus in both groups.

There was also a considerable increase in the ratio of liver weight to body weight in the treated animals compared with the corresponding controls.

*Influence of pyrene.*

No change in the P.N.A.P. : D.N.A.P. ratio was found in the livers of rats maintained on a 5 per cent protein diet and injected with the non-carcinogenic hydrocarbon pyrene (Table V).

TABLE V.—*Animals on 5 per cent Protein Diet and Treated with Pyrene.*

| Rat weight (g.). | Liver weight (g.). | Liver weight percentage of rat weight. | Number of days on diet. | Number of days after injection. | Lipid P mg./100 g. fresh tissue. | T.N.A.P. mg./100 g. fresh tissue. | P.N.A.P. mg./100 g. fresh tissue. | D.N.A.P. mg./100 g. fresh tissue. | P.N.A.P. / D.N.AP. |
|---|---|---|---|---|---|---|---|---|---|
| 127 | 6·72 | 5·30 | 33 | 18 | 116·0 | 113·0 | 89·0 | 24·0 | 3·7 |
| 125 | 5·67 | 4·55 | 42 | 27 | 113·0 | 120·0 | 93·5 | 26·5 | 3·5 |
| 125 | 5·52 | 4·40 | 43 | 28 | 82·2 | 135·0 | 97·0 | 38·0 | 2·6 |
| 135 | 5·31 | 3·95 | 44 | 29 | 105·0 | 118·5 | 94·0 | 24·5 | 3·8 |
| 104* | 5·22 | 5·00 | 47 | 32 | 106·5 | 135·0 | 98·0 | 37·0 | 2·6 |
| Mean | | 4·65 ± 0·6 | .. | .. | 104·5 ± 5·9 | 124·3 ± 4·5 | 94·3 ± 1·6 | 30·0 ± 3·1 | 3·3 ± 0·26 |

* Neutral fat content of liver 9·0 per cent.

There was also no change in the lipid phosphorus concentration or in the liver weight : body weight ratio compared with the control animals on the same diet.

The water and neutral fat contents of the livers of all the animals (treated and control) were, with only three exceptions, within normal limits.

FIG. 1.—Growth rates of individual rats maintained on a 20 per cent protein diet before and after treatment (shown by arrow) with 1:2:5:6-dibenzanthracene (50 mg. in 1 ml. arachis oil per rat intraperitoneal), and P.N.A.P. : D.N.A.P. ratios found for the livers of each rat when killed (k.).. Figures in parentheses give the initial weight (g.) of the rat.

DISCUSSION.

It has been shown that the total nucleic acid content of *adult* animal liver varies with the age and nutritional state of the animal (Rondoni, 1941). In the livers of many species the total nucleic acid *concentration* rises in fasting, but since the liver weight falls as a result of cell volume decrease, there is a fall in the total *amount* of nucleic acid relative to the initial body weight of the animal (Kosterlitz and Cramb, 1943 ; Kosterlitz, 1944*a*, 1944*b* ; Rosenthal and Drabkin, 1943).

Davidson and Waymouth (1944) confirmed this rise in the nucleoprotein phosphorus *concentration* in the liver tissue of fasted adult rats, and also observed a fall in the ratio of pentosenucleic acid to desoxypentosenucleic acid in the tissue. Since the concentration of the latter actually increased, the decrease in the ratio was a result both of this fact and of a concomitant decrease in the pentosenucleic acid (Davidson, 1946).

In our experiments *young* growing rats maintained on a 5 per cent protein diet for a prolonged period also showed a decrease in the liver P.N.A.P. : D.N.A.P. ratio compared with animals maintained on a 20 per cent protein diet. The T.N.A.P. concentration was unchanged, and the decrease in the ratio was the result of an increase in the D.N.A.P. concentration.

In contrast with this " diet effect," administration of 1:2:5:6-dibenzanthracene produced a marked increase in the P.N.A.P. : D.N.A.P. ratio in the livers of rats maintained on either diet. This increase was the result of a considerable decrease in the D.N.A.P. concentration. The livers of both groups of treated rats showed a decrease in T.N.A.P. concentration, and a decrease in P.N.A.P. concentration was also found in the low protein group.

Administration of the non-carcinogenic hydrocarbon pyrene to rats maintained on the 5 per cent protein diet produced no significant change in the nucleic acid balance.

The increase which was found in the liver weight : body weight ratio in the carcinogen-treated rats did not appear to be a result of any increase in the water or neutral fat content of the livers. Kennaway, Kennaway and Warren (1944) have observed a similarly-increased ratio in mice treated with carcinogenic hydrocarbons. Non-carcinogenic substances gave only irregular results. No explanation of this increase in liver weight was advanced. The possibility that the increase is related to a differential inhibitory action of the carcinogen on the growth of the animal body as a whole compared with the growth of the liver is being investigated.

In considering the possible relationship of the decrease in the D.N.A.P. concentration in the livers of the carcinogen-treated rats to growth inhibition and carcinogenesis, it is of interest that Gopal-Ayengar (1947) has shown that in epidermal carcinogenesis, induced experimentally in mice by application of 20-methylcholanthrene, the initial effect of the compound was to produce a decrease in the desoxyribosenucleic acid content of chromosome threads isolated from the epidermal cells. The low desoxyribosenucleic acid content persisted during the precancerous (hyperplastic) stage and was then followed by an increase when malignancy ensued. Stowell (1945) found that X-irradiation of transplanted mouse mammary carcinoma brought about an initial decrease in cellular desoxyribosenucleic acid content, and Mitchell (1942) found a small increase in cytoplasmic pentose nucleotides in biopsied specimens of X-irradiated human tumours. He considered that the effect of the irradiation was to inhibit the conversion of pentosenucleic to desoxypentosenucleic acid.

Such differing agents as carcinogenic compounds and X-radiation, which are capable of producing both inhibition of growth and induction of tumours, thus bring about an initial decrease in the desoxypentosenucleic acid content of the affected cells. It has been shown that the degree of inhibition of rat body growth (Elson and Warren, 1947) and of rat tumour growth (Elson and Haddow, 1947) by 1:2:5:6-dibenzanthracene bears an inverse relationship to the protein content of the diet, and it was suggested that the effect of the carcinogen is to inhibit protein synthesis. Caspersson (1947) has claimed that cytoplasmic protein synthesis is controlled by nucleolus-associated chromatin. Changes in desoxypentosenucleic acid concentration in the cell might thus be expected to result in interference with the synthesis of cytoplasmic proteins. The mechanism of the growth-inhibitory action of 1:2:5:6-dibenzanthracene may therefore be directly connected with this property of increasing the ratio of P.N.A.P. : D.N.A.P. in the cell.

1:2:5:6-Dibenzanthracene does not produce tumours of the liver in rats, but members of a series of derivatives of 4-aminostilbene produce liver tumours in these animals as well as tumours at the site of application (Haddow, Harris, Kon and Roe, 1947). The growth-inhibiting action of these compounds has also been shown to be almost entirely dependent upon the protein content of the diet (Elson and Haddow, 1947). The nucleic acid balance in the livers of rats treated with such substances is being investigated, and thus the effect on the liver nucleic acids of a growth-inhibitory substance which is also a carcinogen for the liver can be followed up to the actual stage of tumour induction.

SUMMARY.

The effect of the administration of 1:2:5:6-dibenzanthracene on the nucleic acid balance in the livers of rats maintained on high and low protein diets has been investigated. The desoxypentosenucleic acid concentration is decreased, and correspondingly there is an increase in the ratio of pentosenucleic to desoxypentosenucleic acid. The implications of this change are discussed in relation to the different growth-inhibitory activity of the carcinogen in animals maintained on diets of different protein content.

The estimations described in the Experimental section were carried out with the assistance of Mr. J. F. Thomas.

This investigation has been supported by grants from the British Empire Cancer Campaign, the Anna Fuller Fund, and the Jane Coffin Childs Memorial Fund, and facilities have also been afforded by Imperial Chemical Industries, Limited. We also wish to thank Miss C. Barrett for technical assistance.

### REFERENCES.

CASPERSSON, T., AND SANTESSON, L.—(1942) *Acta Radiologica*, Suppl. XLVI.

CASPERSSON, T.—(1947) 'Symposia of the Society for Experimental Biology, No. 1: Nucleic Acid.' Cambridge (University Press), p. 127.

DAVIDSON, J. N.—(1946) *J. Physiol.*, **105**, 32P.

*Idem* AND WAYMOUTH, C.—(1944) *Biochem. J.*, **38**, 379.

ELSON, L. A., GOULDEN, F., AND WARREN, F. L.—(1947) *Brit. J. Cancer*, **1**, 80.

*Idem* AND HADDOW, A.—(1947) *Ibid.*, **1**, 97.

*Idem* AND WARREN, F. L.—(1947) *Ibid.*, **1**, 86.
GOPAL-AYENGAR, A.—(1947) *Cancer Res.*, **7**, 1.
HADDOW, A., HARRIS, R. J. C., KON, G. A. R., AND ROE, E. M. F.—(1947) (in press).
KENNAWAY, E. L., KENNAWAY, N. M., AND WARREN, F. L.—(1944) *Cancer Res.*, **4**, 367.
KOSTERLITZ, H. W.—(1944*a*) *Biochem. J.*, **38**, xiv.—(1944*b*) *Nature*, **154**, 207.
*Idem* AND CRAMB, I.—(1943) *J. Physiol.*, **102**, 18P.
MITCHELL, J. S.—(1942) *Brit. J. exp. Path.*, **23**, 296, 309.
RONDONI, P.—(1941) *Schweiz. med. Wchschr.*, **71**, 1354.
ROSENTHAL, O., AND DRABKIN, D. L.—(1943) *J. biol. Chem.*, **150**, 131.
SCHMIDT, G., AND THANNHAUSER, S. J.—(1945) *Ibid.*, **161**, 83.
STOWELL, R. E.—(1945) *Cancer Res.*, **5**, 169.